# Associations between periodontal disease-induced immune cell activation and energy metabolism

**DOI:** 10.3389/fimmu.2026.1773346

**Published:** 2026-03-23

**Authors:** Misaki Iwashita, Fumi Seto-Tetsuo, Akiko Yamashita

**Affiliations:** 1 Department of Periodontology and Endodontology, Nagasaki University Graduate School of Biomedical Sciences, Nagasaki University, Nagasaki, Japan; 2 Department of Microbiology and Oral Infection, Graduate School of Biomedical Sciences, Nagasaki University, Nagasaki, Japan; 3 Department of Periodontology, Division of Oral Rehabilitation, Faculty of Dental Science, Kyushu University, Fukuoka, Japan

**Keywords:** adipose tissue, energy metabolism, inflammation, obesity, periodontitis

## Abstract

Periodontal disease is an infectious-inflammatory disease with widespread systemic effects. Accumulating evidence from clinical and experimental studies has elucidated the mechanisms by which periodontal disease influences systemic energy metabolism, including glucose and lipid metabolisms. These effects are thought to arise from the persistent immune activation associated with periodontitis, leading to systemic inflammatory responses. Specifically, chronic inflammation, such as periodontitis, promotes the infiltration of pro-inflammatory immune cells into adipose tissue, activates immune-metabolic signaling pathways involving dendritic cells, macrophages and T cells, and disrupts thermogenesis and lipid metabolism in adipose tissue and the liver. In addition, periodontal disease has been implicated in alterations in the gut microbiota through the oral-microbiota-gut axis, further contributing to metabolic dysregulation. Collectively, these findings suggest that the influence of periodontal disease on energy metabolism is multifaceted and complex and involves coordinated disturbances in immune regulation, adipose tissue function, hepatic metabolic processes, and gut microbiota homeostasis, ultimately resulting in impaired systemic energy metabolic regulation.

## 1 Introduction

Periodontal disease is highly prevalent worldwide. Chronic inflammation induced by periodontal disease affects not only periodontal tissues, but also the immune system, and can evolve into a state of systemic inflammation. It has been well established through numerous studies conducted worldwide that localized inflammation, such as that observed in periodontal disease, can influence glucose metabolism (1). Obesity is recognized as a major risk factor for type 2 diabetes mellitus and contributes to the development of a wide range of health disorders, including coronary artery disease and metabolic dysfunction-associated steatotic liver disease (MASLD), previously referred to as non-alcoholic fatty liver disease (NAFLD), which is characterized by hepatic steatosis in the presence of metabolic dysfunction. Adipocytes secrete various biologically active molecules, including hormones and cytokines, which are collectively referred to as adipokines. Under physiological conditions, adipocytes predominantly produce anti-inflammatory adipokines that suppress inflammatory responses and play essential roles in the regulation of whole-body metabolic homeostasis. Conversely, obesity is characterized by adipocyte hypertrophy and dysregulated adipokine secretion. Moreover, immune cells, such as macrophages, infiltrate obese adipose tissue, resulting in enhanced production of inflammatory cytokines and chemokines and establishment of a chronic systemic inflammatory state (2, 3). This persistent inflammation constitutes the pathological basis of insulin resistance, atherosclerosis, and related metabolic disorders.

Since the late 1990s, the number of studies investigating the association between periodontal disease and dysregulation of energy metabolism, including lipid metabolism and obesity, has gradually increased worldwide (**Tables 1**, **2**). Numerous epidemiological studies have demonstrated that obesity exacerbates periodontal disease. Recently, several mechanisms have been proposed by which periodontal disease influences lipid metabolism and energy expenditure (4, 5). However, the underlying mechanisms have not been fully elucidated. This review summarizes recent advances in the understanding of the mechanisms by which periodontal disease induces dysregulation of energy metabolism and contributes to the development of obesity, with a focus on the interconnected roles of inflammatory, metabolic, and organ-specific pathways.

**Table 1:** Studies in humans on the association between periodontitis, lipid metabolism and obesity.

Author/Year	Study type	Outcome (s) assessed	Population/Follow-up duration	Key findings	Ref.
Fu/2016	Randomized controlled trial	Effects of periodontal treatment on plasma lipids	109 patients with hyperlipidemia and chronic periodontitis; 2 and 6 months after treatment	Intensive periodontal treatment significantly reduced triglyceride levels and increased HDL-C levels.	(25)
Kato/2018	Retrospective cohort	Effects of obesity on periodontitis progression	2,216 employees of the Electricity Generating Authority of Thailand; 10-year follow-up	After adjustment for confounding factors, obesity was not significantly associated with periodontitis progression (RR 0.98, 95% CI 0.88–1.08)	(45)
Li/2023	Meta-analysis	Prospective longitudinal studies evaluating the association between weight gain and incident periodontitis in adults	Five studies; 42,198 participants	Overweight and obese individuals had a higher risk of incident periodontitis (RR 1.13, 95% CI 1.06–1.20; RR 1.33, 95% CI 1.21–1.47, respectively) compared with individuals who maintained normal weight	(21)
Miyauchi/2025	Intervention	Comprehensive analysis of salivary and gut microbiota by 16S rRNA sequencing and serum metabolite profiling	23 patients with Stage III, Grade B periodontitis; samples collected before and ≥1 month after treatment	High alpha diversity in salivary and gut microbiota; distinct gut microbiota composition compared with healthy controls at baseline; concordant changes in gut microbiota and serum metabolic profiles; improvement of salivary microbiota following periodontal treatment Improvement of salivary microbiota with periodontal disease treatment	(47)
Munenaga/2013	Longitudinal	BMI and WC at ages 31 and 46; periodontal examination at age 46	725 never-smoking participants assessed at ages 31 and 46	Obesity, central obesity, and weight gain were associated with a greater number of sites with PPD ≥4 mm and bleeding PPD ≥4 mm	(13)
Nescimento/2015	Cross-sectional	Associations between periodontal status and lipid profiles	118 medical staff at an outpatient clinic in Tokyo	Periodontal inflammation was inversely associated with HDL-C, particularly in females.	(23)
Saito/2024	Cross-sectional	Associations between periodontal status and lipid profiles	5,342 participants from the 2009-2014 NHANES	Elevated TG, TC, and LDL-C levels were positively associated with severe periodontitis; high TC, LDL-C, and TG groups showed increased odds of severe periodontitis (OR 1.55, 95% CI 1.17–2.05; OR 1.50, 95% CI 1.09–2.06; OR 1.35, 95% CI 1.02–1.79, respectively)	(24)
Tegelberg/ 2021	Longitudinal	Association between periodontal status and incidence of MetS	4,761 participants undergoing health examinations; 8-year follow-up	Presence of periodontal pockets ≥6 mm was associated with a higher risk of MetS onset (RR 1.30, 95% CI 1.01–1.67) and abdominal obesity (RR 1.25, 95% CI 1.01–1.56)	(22)
Yoneda/2012	Intervention	Detection frequency of periodontal bacteria in oral samples and serum AST/ALT levels following non-surgical periodontal therapy (3 months)	150 NAFLD and 60 non-NAFLD controls	Higher prevalence of *P. gingivalis* in NAFLD patients than in controls (46.7% vs. 21.7%; OR 3.16); improvement in serum AST and ALT levels after periodontal treatment in NAFLD patients with periodontitis	(42)
Yoshida/ 2022	Cross-sectional	Correlation between IgG antibody titers against periodontal pathogens and clinical/biochemical parameters	52 patients with NAFLD	Anti-*A. actinomycetemcomitans* IgG titers were positively correlated with visceral fat, fasting plasma insulin, and HOMA-IR, and negatively correlated with the liver-to-spleen ratio	(41)

AST, aspartate aminotransferase; ALT, alanine aminotransferase; NAFLD, non-alcoholic fatty liver disease; HDL-C, high-density lipoprotein cholesterol; RR, relative risk; CI, confidence interval; BMI, body mass index; WC, waist circumference; PPD, probing pocket depth; NHANES, national health and nutrition examination survey; TG, triglyceride; TC, total cholesterol; LDL-C, low-density lipoprotein cholesterol; OR, odds ratio; MetS, metabolic syndrome; HOMA-IR, homeostasis model assessment−insulin resistance.

**Table 2:** Studies in animals on the association periodontitis and lipid metabolism and obesity.

Author/Year	Animal/Treatment	Key findings	Ref.
Chen/2025	C57BL/6 and Ahr^−/−^ mice; ligation (6 weeks)	Promotion of MASLD; gut microbiota dysbiosis; depletion of tryptophan metabolism	(40)
Dong/2022	C57BL/6 mice; oral administration of *P. gingivalis* (twice weekly for 6 weeks)	Increased fat mass and impaired glucose tolerance; enhanced adipose tissue inflammation and intestinal permeability; compositional and functional alterations of the gut microbiota	(4)
Dos/2017	Wistar rats; ligation-induced periodontitis (20 days) followed by ligature removal	Reduction in steatosis score; increased glutathione levels; decreased malondialdehyde, total cholesterol, and triglyceride concentrations after ligature removal	(44)
Fujita/2018	Wistar rats; oral administration of *P. gingivalis* LPS (single or daily for 10 days), HFD	Development of NASH (macrovesicular steatosis, hepatocyte ballooning, and inflammatory cell infiltration); increased and prolonged hepatic accumulation of *P. gingivalis* LPS	(43)
Hatasa/2021	C57BL/6 mice; intravenous injection of *P. gingivalis* (single dose, 18 hours)	Downregulation of cholesterol homeostasis and PI3K/Akt/mTOR signaling gene sets in BAT; altered endocrine function of BAT	(36)
Kashiwagi/2021	db/db mice; oral administration of *P. gingivalis* (every 3 days for 30 days)	Altered gut microbiome profiles and intestinal metabolite levels; enterohepatic metabolic derangements; exacerbation of hyperglycemia	(46)
Kato/2018	C57BL/6 mice; oral administration of *P. gingivalis* (twice weekly for 5 weeks)	Altered gut microbiota; elevated amino acid profiles associated with increased risk of diabetes and obesity	(45)
Komazaki/2017	C57BL/6 mice; oral administration of *A. actinomycetemcomitans* (6 times weekly for 6 weeks), HFD	Altered gut microbiota composition; upregulation of fatty acid biosynthesis; downregulation of fatty acid degradation acid degradation	(5)
Li/2025	C57BL/6 mice; ovariectomized; ligation and oral administration of *P. gingivalis* (every other day for 4 weeks)	Increased M1-polarized macrophages within the visceral adipose tissue of OVX mice with periodontitis. Small EVs from M1-polarized macrophages promoted systemic inflammation by delivering pro-inflammatory miRNAs.	(38)
Liu/2024	C57BL/6 mice; oral administration of *P. gingivalis* and *F. nucleatum* (3 times weekly for 14 weeks)	Reduced AKT phosphorylation, adiponectin, leptin, and expression of genes related to adipogenesis and lipogenesis in WAT; gingival EVs derived from polymicrobial oral infection–induced dysfunction of WAT	(37)
Sasaki/2018	C57BL/6 mice; intravenous injection of *P. gingivalis* (twice weekly for 12 weeks), HFD	Increased visceral and subcutaneous fat mass; enrichment of fatty acid metabolism, hypoxia, and TNF-α/NF-κB signaling pathways; altered gut microbiota and reduced bacterial diversity	(35)
Sun/2025	Wister rats; ligation (8 weeks)	Exacerbation of hepatic lipid accumulation, fibrosis, and oxidative stress	(39)
Yoneda/2012	C57BL/6 mice; intravenous injection of *P. gingivalis*, HFD	Acceleration of NAFLD progression in a murine NAFLD model	(42)
Yoshida/2022	Pregnant C57BL/6 mice; oral and intravenous administration of *P. gingivalis* (3 times weekly; total of 8 administrations)	Obesity; altered hepatic lipid metabolism; modified gene expression in the liver and BAT; reduced fetal weight.	(41)
Wu/2023	Sprague–Dawley rats; ligation (3 months) Visceral adipocytes from rats stimulated with *P. gingivalis* LPS	Hypoadiponectinemia; increased GRP78 and IRE1α phosphorylation in visceral adipocytes Increased GRP78 and p-IRE1α/IRE1α levels; decreased adiponectin protein	(34)

MASLD, metabolic dysfunction-associated steatotic liver disease; LPS, lipopolysaccharide; NASH, non-alcoholic steatohepatitis; BAT, brown adipose tissue, HFD, high fat diet; OVX, ovariectomy; EV, extracellular vesicle; WAT, white adipose tissue; NAFLD, non-alcoholic fatty liver disease.

## 2 Systemic dissemination of periodontal inflammation

In periodontal tissues affected by periodontitis, immune cells including dendritic cells, macrophages, neutrophils, and Th1/Th17 cells are persistently activated by periodontal pathogens and their endotoxins. These activated cells produce a variety of proinflammatory cytokines, such as interferon-γ, interleukin (IL)-1β, IL-6, and tumor necrosis factor-α (6). Although periodontitis is primarily a localized inflammatory disease of the oral cavity, accumulating evidence indicates that inflammatory mediators and bacterial components can enter systemic circulation, thereby inducing systemic inflammation. When periodontal inflammation becomes chronic, these inflammatory factors are continuously released into the bloodstream, potentially disrupting systemic immune homeostasis (7). Given the chronic nature of periodontitis and the frequent occurrence of bacteremia, sustained systemic exposure to endotoxins and inflammatory mediators is considered pathologically significant.

A growing body of evidence suggests that periodontal inflammation enhances the systemic innate immune responses. Peripheral blood neutrophils and monocytes isolated from patients with periodontitis exhibit exaggerated inflammatory cytokine production in response to bacterial stimulation or endotoxin exposure compared with those from healthy individuals (8). In an experimental mouse model of periodontitis, Yu et al. demonstrated that chronic inflammatory conditions could reprogram innate immune cells in the bone marrow through trained immunity, thereby exacerbating ongoing inflammation and increasing susceptibility to other inflammatory diseases (9). Consistent with this concept, *Porphyromonas gingivalis* (*P. gingivalis*) induces long-lasting activation, or trained immunity, in human monocytes (10). Moreover, periodontitis has been reported to promote the production and activation of neutrophils in the bone marrow, further contributing to the pathogenic interplay between periodontitis and systemic inflammatory diseases (11).

Metabolic conditions such as obesity further amplify the systemic impact of periodontal inflammation. Nakarai et al. reported that the administration of low-dose lipopolysaccharide (LPS) to genetically and diet-induced obese mice led to marked increases in serum amyloid A, C-C motif ligand (CCL) 5, and serum LPS-binding protein (LBP) levels compared with non-obese control mice (12). These findings suggest that periodontal inflammation is more likely to exert systemic effects in obese individuals than in those with a normal weight. Supporting this notion, an interventional study in patients with diabetes and periodontitis demonstrated that individuals with a body mass index (BMI) of approximately 25 kg/m² exhibited elevated baseline levels of high-sensitivity C-reactive protein (CRP) exceeding 500 ng/mL, which is indicative of heightened systemic inflammation. In this subgroup, periodontal treatment significantly reduced CRP levels and was accompanied by improvements in glycated hemoglobin (HbA1c). In contrast, individuals with a BMI of approximately 23 kg/m² showed lower baseline CRP levels (<500 ng/mL) and did not exhibit significant changes in CRP or HbA1c levels following periodontal therapy (13). Collectively, these findings suggest that in individuals with mild obesity (BMI approximately 25 kg/m²), periodontal inflammation is more readily amplified by adipose tissue and disseminated systemically than in individuals with normal weight. Because immune cells within obese adipose tissue are already in a primed or activated state, periodontal inflammation in obese patients is likely to be further exacerbated, ultimately exerting a substantial impact on systemic immune homeostasis. Therefore, periodontal treatment may play an important role in attenuating obesity-associated systemic inflammatory burden.

## 3 Regulation of energy metabolism

Energy homeostasis is maintained by an integrated regulatory network that coordinates feeding behavior, energy expenditure, and nutrient metabolism. Dietary fat contributes directly to lipid stores, while excess energy is also converted into lipids through *de novo* lipogenesis, primarily in the liver and adipose tissue, and subsequently stored in adipocytes. In obesity, adipocyte hypertrophy limits further lipid storage capacity, resulting in increased release of free fatty acids (FFAs) into the circulation. Elevated circulating FFAs are taken up by peripheral tissues such as skeletal muscle and the liver, where they are predominantly stored as triglycerides, promoting ectopic lipid accumulation and lipotoxicity (14).

At the cellular level, nutrient-sensing signaling pathways, including AMP-activated protein kinase (AMPK) and mechanistic target of rapamycin (mTOR), play central roles in regulating lipid and glucose metabolism in response to intracellular energy status (15, 16). AMPK, in particular, is activated under conditions of energy deprivation and restores energy balance by enhancing fatty acid oxidation while suppressing anabolic processes such as gluconeogenesis.

In recent years, gut microbiota has emerged as a key regulator of host energy homeostasis. Microbiota-derived metabolites, particularly short-chain fatty acids (SCFAs) and tryptophan-derived metabolites, modulate host energy metabolism by influencing intestinal hormone secretion as well as immune and inflammatory pathways (17, 18). Disruption of the coordinated interactions among central regulatory circuits, peripheral metabolic tissues, and the intestinal environment can therefore contribute to the development of metabolic disorders, including obesity and MASLD. SCFAs, which are produced in high concentrations through fermentation of dietary fiber by intestinal bacteria, play a central role in regulating host energy utilization and storage (19). Moreover, the functional state of the gut microbiota, especially its capacity to produce SCFAs, is closely linked to intestinal pH, barrier integrity, immune responses, and inflammatory status, thereby exerting broad effects on whole-body glucose and lipid metabolism and insulin sensitivity (20).

## 4 Clinical evidence on the effects of periodontal disease on energy metabolism

Epidemiological studies have examined the association between periodontal disease and obesity have been conducted worldwide, with many focusing on the impact of obesity on periodontal health. To date, many studies have shown that obesity exacerbates periodontal disease. For example, a recent Mendelian randomization analysis based on genome-wide association study data from European populations demonstrated a causal relationship, in which increases in adult BMI and body fat percentage elevated the risk of periodontal disease (21). This association is further supported by several long-term longitudinal studies. Tegelberg et al. reported that individuals who were not abdominally obese at 31 years of age but developed abdominal obesity by 46 years of age exhibited a higher relative risk of an increased number of periodontal pockets ≥4 mm and sites with bleeding on probing (22). In addition, a meta-analysis of longitudinal studies with follow-up periods exceeding three years showed that individuals who became overweight or obese during the observation period had a significantly higher risk of developing periodontitis compared with those who maintained normal body weight (23).

In contrast to the abundance of research supporting obesity-induced periodontal deterioration, considerably fewer studies have examined the reverse relationship, namely, whether periodontal disease itself contributes to obesity and systemic energy metabolism. However, emerging evidence, particularly from basic and translational research, has increasingly suggested a potential role for periodontal inflammation in metabolic dysregulation. At present, clinical evidence directly addressing this issue remains limited but is suggestive. An 8-year prospective cohort study investigating the relationship between periodontitis and the development of metabolic syndrome demonstrated that individuals with periodontal pockets ≥6 mm had a significantly higher relative risk of developing abdominal obesity compared with those with periodontal pockets ≤3 mm (24). This finding implies that periodontal disease may precede and contribute to obesity-related metabolic abnormalities, rather than merely coexisting with them. Furthermore, a randomized controlled trial assessing blood lipid profiles before, two months after, and six months after periodontal treatment in patients with hyperlipidemia and chronic periodontitis reported a significant reduction in triglyceride levels and a significant increase in high-density lipoprotein cholesterol compared with baseline. These results indicate that periodontal treatment may beneficially modulate systemic lipid profiles, suggesting the potential influence of periodontal disease on lipid metabolism (25). In addition to these clinical observations, transcriptome analysis using single-cell RNA sequencing has recently provided further insight. A comparative analysis of cells derived from periodontitis patients and healthy controls revealed enhanced lipid metabolism–related gene expression in immune cell populations, particularly in mast cells, in the periodontitis group (26). While this finding was derived from ex vivo analyses, they provide mechanistic support for a link between periodontal inflammation and altered lipid metabolic pathways. Although the number of clinical studies directly addressing the impact of periodontal disease on obesity and energy metabolism remains limited, available evidence suggests that periodontal disease may act as a potential upstream factor in systemic metabolic abnormalities. These findings support the notion that periodontal disease itself may affect lipid metabolism and obesity-related phenotypes, and further, well-designed clinical and intervention studies are warranted to clarify its causal relationship and underlying mechanisms.

## 5 Mechanisms by which periodontal disease affects energy metabolism

### 5.1 Effects on adipose tissue

Obesity is characterized by macrophage-driven adipose tissue inflammation that enhances adipokine production (3, 4), and this inflammatory state may be further exacerbated in obese patients with periodontitis. Based on this concept, a previous study investigated whether immune cells activated by periodontal infection infiltrated visceral adipose tissue and promoted inflammation using a co-culture model of adipocytes and macrophages. In adipocytes co-cultured with macrophages in the presence of low concentrations of LPS, marked upregulation of gene expression has been observed for numerous inflammatory cytokines and chemokines, including CCL5, CCL19, IL-6, and monocyte chemoattractant protein-1 (27, 28). CCL19 is a small cytokine of the CC family, also known as MIP-3-beta or ELC, highly expressed in the thymus, lymph nodes, and activated bone marrow stromal cells. CCL19 signals through G protein-coupled receptor C–C chemokine receptor 7 (CCR7) to control a diverse array of migratory events in adaptive immunity following antigen encounter with immunocytes (29).

In obese individuals, increased numbers of CCR7-expressing cells in adipose tissue and elevated circulating CCL19 levels have been reported, along with positive correlations between adipose tissue CCL19 expression and BMI, as well as circulating levels of CCL5, CCL19, and IL-12 (30, 31). To further investigate the functional significance of CCL19, transgenic mouse models with adipocyte-specific overexpression of CCL19 were developed and used to examine the effects of CCL19 signaling on metabolic regulation *in vivo*. This study demonstrated that activation of the CCL19/CCR7 pathway in adipose tissue promotes inflammatory responses and suppresses AMPKα through ERK1/2 signaling, thereby disrupting lipid metabolism, energy homeostasis, and thermogenic processes, ultimately resulting in increased adipose tissue accumulation (32). Collectively, these findings suggest that inflammation induced by periodontal disease may disseminate systemically via adipose tissue and influence energy homeostasis by activating immune cell responses in adipose tissue, including the CCL19/CCR7 pathway (32, 33). In animal models administered periodontal pathogens or endotoxins, induction of endoplasmic reticulum stress in adipocytes has been observed (34), indicating that exposure to periodontal infection–related factors can impair adipocyte cellular function, essentially placing fat cells under physiological stress that disrupts their normal metabolic activities. Sasaki et al. showed that the administration of *P. gingivalis* to high-fat diet–fed mice resulted in greater increases in body weight and adipose tissue mass than in non-treated controls (35). Other studies have suggested that *P. gingivalis*-induced endotoxemia alters the endocrine function of brown adipose tissue, thereby contributing to obesity (36). Experimental models of long-term infection with multiple periodontal pathogens have demonstrated the development of white adipose tissue (WAT) dysfunction, characterized by reduced AKT phosphorylation, decreased adiponectin and leptin levels, and downregulation of genes involved in adipogenesis and lipogenesis (37). This WAT dysfunction has been shown to be mediated, at least in part, by gingival cell–derived extracellular vesicles (EVs). In addition, Li et al. reported an increased accumulation of M1-polarized macrophages in the visceral adipose tissue of ovariectomized mice with periodontitis, and demonstrated that small EVs derived from M1-polarized macrophages promote systemic inflammation through the delivery of pro-inflammatory microRNAs (38). These findings by Li et al. suggest that macrophage-mediated mechanisms observed in periodontitis, particularly in postmenopausal women, may provide a foundation for the development of novel therapeutic strategies targeting both periodontitis and systemic inflammatory diseases, provided that the mechanisms underlying individual susceptibility and dysregulation can be more fully elucidated.

### 5.2 Effects on the liver

In a rat molar ligation-induced experimental periodontitis model, downregulation of hepatic fatty acid β-oxidation-related enzymes was associated with increased hepatic lipid accumulation and aggravated NAFLD (currently termed MASLD) (39). Chen et al. demonstrated that periodontitis exacerbates MASLD by disrupting the gut microbiota–tryptophan metabolism–aryl hydrocarbon receptor (AhR) axis (40). They further showed that supplementation with tryptophan-derived indole-3-propionic acid or the tryptophan-metabolizing probiotic *Limosilactobacillus reuteri* attenuated these adverse effects in an AhR-dependent manner. Although intestinal tryptophan metabolism proceeds via multiple pathways, the microbiota-driven indole pathway accounts for a substantial proportion of luminal tryptophan catabolism and represents the primary physiological source of AhR ligands. Collectively, these findings indicate that the disruption of the indole pathway has a disproportionate effect on host metabolic and inflammatory homeostasis, thereby contributing to the exacerbation of MASLD. Moreover, administration of *P. gingivalis* in mice has been shown to induce alterations in the expression of lipid metabolism–related genes in both the liver and adipose tissue, characterized by upregulation of the interleukin-6/Janus kinase/signal transducer and activator of transcription 3 (IL-6/JAK/STAT3) signaling pathway and downregulation of genes involved in fatty acid metabolism (41). In a mouse model of high-fat diet–induced NAFLD (currently termed MASLD), intravenous administration of *P. gingivalis* increased the body and liver weights, serum alanine aminotransferase levels, and hepatic triglyceride content (42). Fujita et al. reported that hepatic accumulation of *P. gingivalis* lipopolysaccharide is increased and persists for longer durations in fatty livers compared with normal livers (43). Importantly, microvesicular steatosis induced by experimental periodontitis in rats was ameliorated following removal of the ligature (44). Furthermore, periodontal treatment in patients with NAFLD (currently termed MASLD) has been shown to improve serum aspartate aminotransferase and alanine aminotransferase levels (42). Collectively, these findings suggest that periodontitis contributes to the development and progression of fatty liver disease and may represent an independent risk factor for MASLD.

### 5.3 Effects on intestinal environment

Periodontal pathogens are continuously ingested with saliva. Under physiological conditions, gastric acid and the intestinal environment effectively limit bacterial survival and colonization. In periodontitis, however, increased bacterial load and enhanced bacterial survival facilitate the translocation of oral bacteria into the intestinal tract, which can lead to gut microbiota dysbiosis. Importantly, this process is closely associated with intestinal barrier dysfunction, allowing microbial components and metabolites to translocate systemically, thereby influencing metabolic organs. In mice orally administered the periodontal pathogen *P. gingivalis*, significant alterations in gut microbiota composition have been observed, accompanied by increases in serum metabolites associated with an elevated risk of diabetes and obesity (5, 45). In obese and diabetic mouse models, oral *P. gingivalis* administration similarly induced gut dysbiosis and intestinal metabolite changes, and exacerbated metabolic abnormalities including hyperglycemia (46). Dong et al. further demonstrated that *P. gingivalis*–treated mice exhibit increased intestinal permeability, adipose tissue inflammation, increased fat mass, and impaired glucose tolerance (4). Collectively, these findings highlight the importance of intestinal barrier dysfunction as a mechanistic link between periodontal disease, adipose tissue inflammation, metabolic dysregulation, and liver disease.

In humans, periodontitis has also been associated with altered gut microbiota composition and serum metabolite profiles (47), supporting a link between periodontal disease–related gut dysbiosis and systemic metabolic alterations. Moreover, the salivary microbiome differs between obese and normal-weight individuals, with obesity-related profiles showing higher immune-related signatures and reduced functional capacity for environmental adaptation and xenobiotic degradation (48). These observations suggest a bidirectional interaction between the oral microbiota and obesity, although further studies are needed to clarify the mechanisms by which oral microbes influence susceptibility to systemic inflammatory and metabolic diseases.

## 6 Limitations and future directions

As patients with diabetes, a representative disease closely associated with periodontitis, frequently present with obesity, careful selection of appropriate study designs and analytical methods is required to determine whether obesity is independently associated with periodontal disease. Based on a 10-year cohort study, Charupinijkul et al. argued that obesity was not an independent risk factor for periodontitis progression (49). However, they noted several limitations of their study, including potential bias in the socioeconomic status of the participants and lack of information regarding prior periodontal treatments. In addition, differences in participant selection, ethnicity, age, and sex may have contributed to discrepancies in the study results.

Traditionally, BMI has been used as a clinical parameter for defining obesity. However, recent studies have questioned the suitability of BMI as a true indicator of obesity, because it depends solely on body weight and height. Alternative indices of obesity, such as waist circumference and visceral fat area, have been proposed, and the advantages and limitations of these measures in analyzing the associations between obesity and other diseases have been widely discussed. Iwai et al. reported that periodontal pocket depth ≥4 mm was significantly associated with an elevated body shape index (ABSI) ≥0.0080, whereas no significant association was observed with BMI ≥25 kg/m² (50). They suggested that ABSI may represent a more sensitive indicator of obesity than BMI when examining its association with periodontal disease. Furthermore, a cross-sectional study conducted in the United States (51) reported that the Conicity Index (C-index), an indicator of abdominal obesity, was independently associated with the prevalence of periodontitis. Notably, the C-index demonstrated a significantly superior predictive performance for periodontitis compared with conventional measures, such as BMI and waist circumference, suggesting its potential utility as a sensitive marker for the early detection and assessment of periodontitis.

Although animal models are valuable for elucidating causal relationships and for the initial evaluation of therapeutic interventions, they cannot fully recapitulate the complex physiological and immunological environments of humans. Therefore, findings from animal studies should be interpreted with caution and validated in clinical settings. Particular care is warranted when interpreting studies involving the administration of periodontal pathogens or endotoxins, as such models typically introduce only a limited subset of key pathogens and do not reproduce the diverse and complex polymicrobial biofilm characteristics of human periodontitis. Moreover, anatomical and physiological differences between species can result in substantial variations in disease progression, immune responses, and treatment outcomes (52). Experimental variables, including administration routes, exposure duration, bacterial strains, and species, may further influence the study results. In addition, as discussed above, potential alterations in the gut microbiota induced by the oral or intravenous administration of periodontal pathogens should be carefully considered.

Finally, although longitudinal data examining the relationship between periodontal disease and energy metabolism are gradually accumulating, the current body of evidence remains insufficient to draw definitive conclusions. Therefore, future research should prioritize well-designed, long-term interventional studies with rigorous follow-up to establish more robust evidence.

## 7 Conclusion

**Figure 1:**
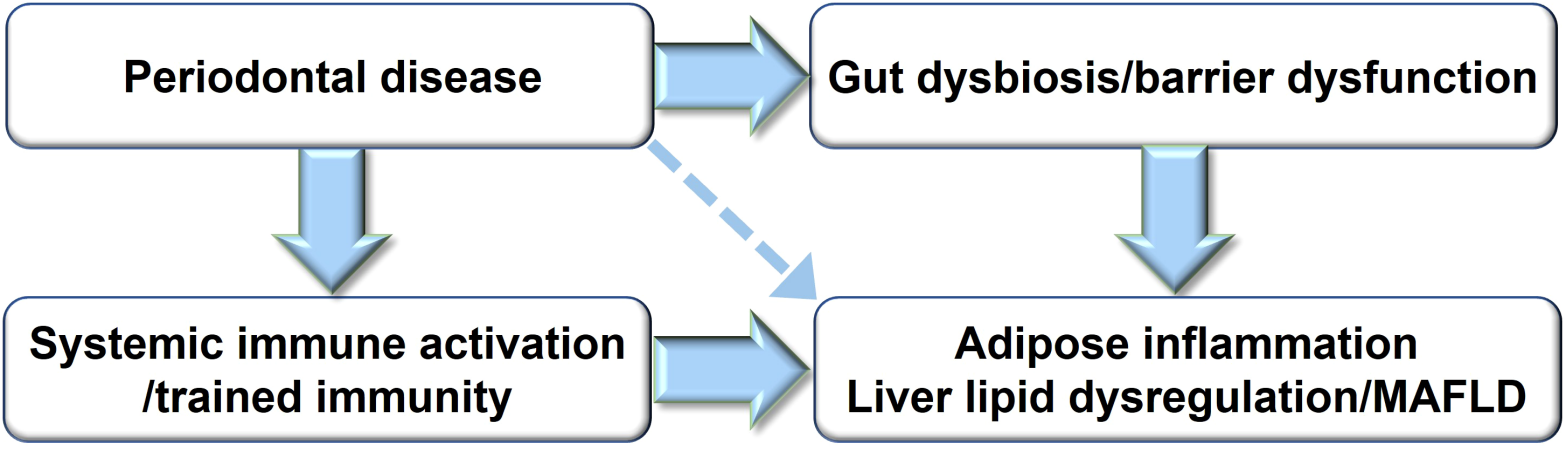
Mechanisms by which periodontal disease affects immunometabolic imbalance. MASLD, metabolic dysfunction-associated steatotic liver disease.

**Figure 2:**
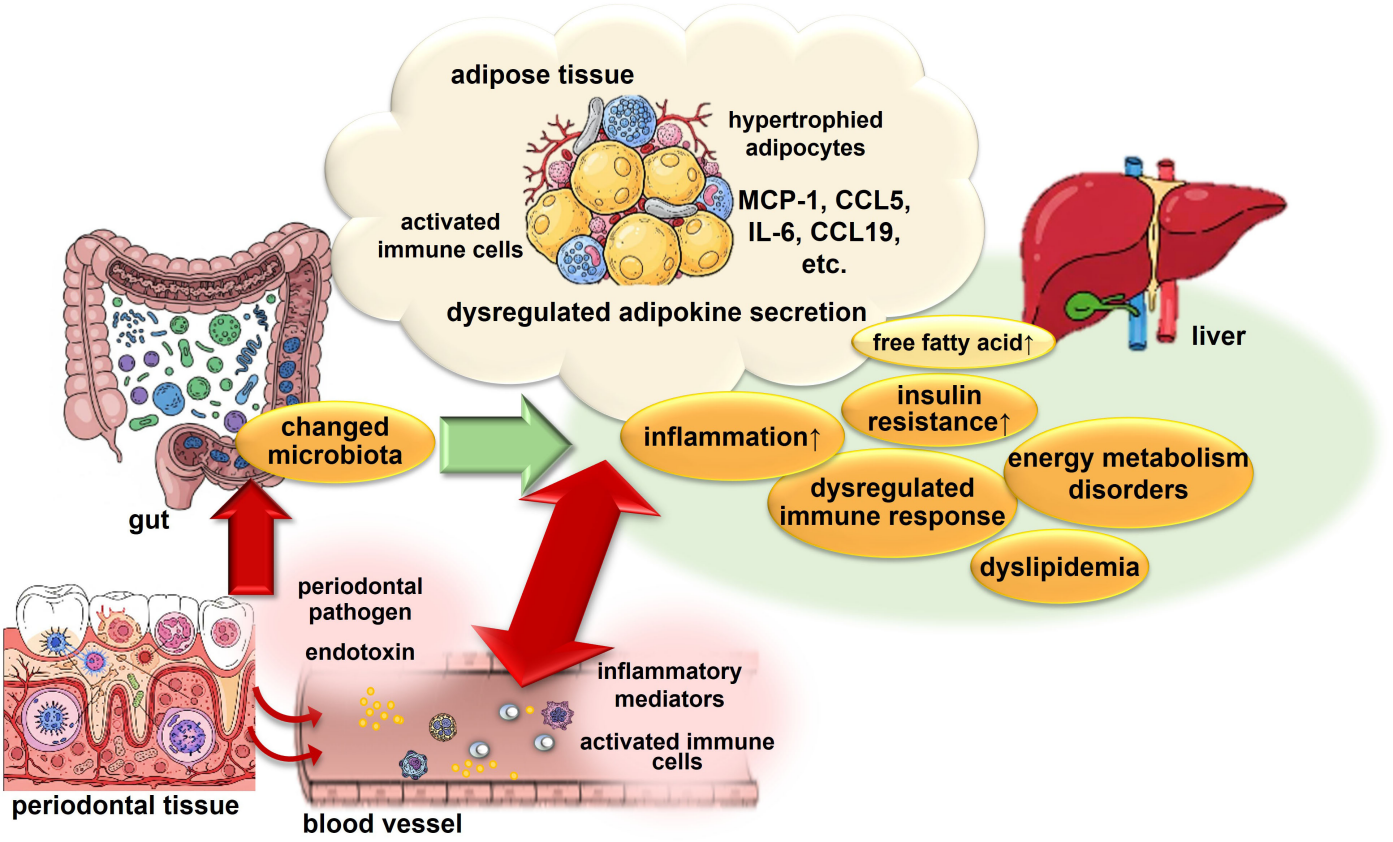
Schematic diagram of the mechanisms related to periodontal disease and energy metabolism.

Multiple mechanisms have been implicated in the effects of periodontal disease on energy metabolism, including chronic activation of immune cells induced by periodontitis, infiltration of inflammatory immune cells into adipose tissue, activation of immunometabolic pathways, such as the CCL19/CCR7 axis, dysregulation of lipid metabolism and thermogenesis in adipose tissue and the liver, and gut microbiota dysbiosis mediated through the oral-microbiota-gut axis. These mechanisms are neither linear nor isolated. Rather, periodontal disease appears to induce abnormalities in energy metabolic regulation by integratively disrupting the immune responses, adipose tissue function, hepatic metabolism, and gut microbiota (**Figures 1**, **2**).
